# Issues materno-fœtales des grossesses non suivies à Lubumbashi, République Démocratique du Congo

**DOI:** 10.11604/pamj.2019.33.66.18528

**Published:** 2019-05-29

**Authors:** Amani Maleya, Yves Kalume Kakudji, Roger Munan Mwazaz, Joseph Bulenda Nsambi, Hugues Ilunga Ngwej, Olivier Mukuku, Xavier Kinenkinda, Prosper Kakudji Luhete

**Affiliations:** 1 Département de Gynécologie-Obstétrique, Faculté de Médecine, Université de Lubumbashi, République Démocratique du Congo; 2 Institut Supérieur des Techniques Médicales de Lubumbashi, République Démocratique du Congo

**Keywords:** Grossesse non suivie, consultations prénatales, pronostic maternel et périnatal, Lubumbashi, Pregnancy not followed, antenatal care, maternal and perinatal outcomes, Lubumbashi

## Abstract

**Introduction:**

Les grossesses non suivies se caractérisent par leur morbi-mortalité et maternelle et fœtale importante. Cette étude s'est fixée comme objectifs de déterminer la fréquence des grossesses non suivies, décrire le profil sociodémographique et évaluer la morbi-mortalité materno-fœtale lors de l'accouchement chez les femmes n'ayant pas suivi de consultations prénatales (CPN) dans la ville de Lubumbashi.

**Méthodes:**

Une étude transversale analytique des accouchées d'une grossesse monofoetale de décembre 2013 à mai 2014 a été mené dans 10 maternités de référence à Lubumbashi. Les femmes n'ayant pas suivi les CPN ont été comparées aux femmes qui les avaient bien suivies (CPN≥4). Les paramètres sociodémographiques maternels, la morbi-mortalité maternelle et périnatale ont été analysées. L'odds ratio et son intervalle de confiance ont été calculés. Le seuil de signification a été fixé à une valeur de p<0,05.

**Résultats:**

Nous avons trouvé que la fréquence de l'absence de suivi de grossesses était de 21,23% et le nombre moyen de consultations prénatales était de 2,6 ± 1,9. L'analyse de la relation entre les consultations prénatales et les caractéristiques sociodémographiques des accouchées montre que l'absence de suivi était 2,29 fois plus élevée chez les adolescentes que chez les femmes adultes (OR=2,29 [1,54-3,41]), 4 fois plus élevée chez les femmes vivant seules que chez celles vivant en union (OR=4,00 [2,05-7,79]) et 4,08 fois plus élevée chez les femmes de bas niveau de scolarité (analphabète ou primaire) que chez celles ayant un niveau de scolarité élevé (OR=4,08 [3,08-5,40]). Comparées à celles les ayant bien suivis, nous avons constaté que les femmes n'ayant pas suivi de consultations prénatales présentaient un risque élevé d'évacuation obstétricale (OR=1,90 [1,26-2,95]), de rupture de membranes fœtales à l'admission (OR=1,31 [1,02-1,68]), de mal présentation fœtale (OR=1,89 [1,03-3,44]), d'accouchement par césarienne (OR=1,78 [1,21-2,63]), d'éclampsie (OR=3,00 [1,09-8,70]), de rupture utérine (OR=4,76 [1,00-47,19]) et d'anémie (OR=2,33 [1,06-5,13]). Les taux de prématurité (OR=1,93 [1,33-2,80]), de post-maturité (OR=1,47 [1,00-2,30]), de faible poids de naissance (OR=2,33 [1,56-3,46]), de dépression néonatale (OR=3,89 [2,52-6,02]), de transfert en néonatologie (OR=1,60 [1,11-2,32]) et de mortalité périnatale (OR=2,70 [1,59-4,57]) étaient significativement plus élevés chez les nouveau-nés issus des femmes n'ayant suivi de consultations prénatales que chez ceux de celles les ayant bien suivis.

**Conclusion:**

Il ressort de notre étude que l'absence de suivi des consultations prénatales est associée à une forte morbidité maternelle et une morbi-mortalité périnatale élevée dans notre milieu.

## Abstract (en)

**Introduction:**

Unattended pregnancies are characterized by significant morbidity maternal and morbidity and mortality fetal. This study aims to determine the frequency of unattended pregnancies, to describe the socio-demographic profile and to assess the maternal and fetal outcomes during childbirth among women who did not attend antenatal care (ANC) in the city of Lubumbashi.

**Methods:**

It was an analytical cross-sectional study of maternal pregnancy from December 2013 to May 2014 in 10 maternity hospitals in Lubumbashi. Women who did not receive ANC were compared to women who had followed them (ANC≥4). Maternal socio-demographic parameters, maternal and perinatal morbidity and mortality were analyzed.

**Results:**

We found that the frequency of no pregnancy monitoring was 21.23% and the mean number of antenatal visits was 2.6 ± 1.9. An analysis of the relationship between ANC and socio-demographic characteristics of women who were delivered shows that the lack of follow-up was 2.29 times higher for adolescent girls than for adult women (OR=2.29 [1.54-3.41]), 4 times higher for women living alone than for women living in unions (OR=4.00 [2.05-7.79]) and 4.08 times higher for women with low levels of education (illiterate or primary) than among those with a high level of education (OR=4.08 [3.08-5.40]). Compared to those who followed them well, we did not find that women who did not attend ANC had a high risk of obstetric emergency (OR = 1.90 [1.26-2.95]), rupture of membranes fetal admission (OR=1.31 [1.02-1.68]), fetal mal presentation (OR=1.89 [1.03-3.44]), caesarean delivery (OR=1.78 [1.21-2.63]), eclampsia (OR=3.00 [1.09-8.70]), uterine rupture (OR=4.76 [1.00-47.19]) and anemia (OR=2.33 [1.06-5.13]). Rates of preterm birth (OR = 1.93 [1.33-2.80]), post-maturity (OR=1.47 [1.00-2.30]), low birth weight (OR=2.33 [1.56-3.46]), neonatal depression (OR=3.89 [2.52-6.02]), neonatal transfer (OR=1.60 [1.11-2.32]) and perinatal mortality (OR=2.70 [1.59-4.57]) were significantly higher in neonates from women with prenatal consultations than in those with well followed.

**Conclusion:**

Our study shows that the lack of follow-up of ANC is associated with high maternal morbidity and high perinatal morbidity and mortality in our environment.

## Introduction

La grossesse est un événement naturel et physiologique particulier qui ne se déroule pas toujours normalement et est responsable d'une morbidité et d'une mortalité évitables. Dans le monde, 830 femmes environ meurent chaque jour de causes évitables liées à la grossesse et à l'accouchement. En 2015, 303 000 femmes sont décédées pendant ou après la grossesse ou l'accouchement. La majeure partie de ces décès se sont produits dans des pays à revenu faible et la plupart auraient pu être évités [1, 2]. En République Démocratique du Congo (RDC), selon les récentes estimations, le ratio de mortalité maternelle s'établit à 846 décès pour 100 000 naissances vivantes et le taux de mortalité néonatale à 28‰ [3]. Son suivi est alors nécessaire afin d'identifier d'éventuels risques et d'améliorer le pronostic de la grossesse [4]. Les soins prénataux ont été adoptés de façon universelle comme une pratique médicale qui s'organise autour d'un ensemble des gestes techniques simples mais rigoureux aboutissant à trois objectifs essentiels: (i) vérifier le bon déroulement de la grossesse et dépister tous les facteurs de risque antérieurs et contemporains, (ii) traiter ou orienter la femme, les cas échéants vers les surveillances ou une thérapeutique spécialisée en raison d'un facteur de risque décelé et (iii) établir le pronostic de l'accouchement, prévoir les conditions d'accouchement de manière à ce que toutes les dispositions pratiques soient prises pour éviter les évènements dangereux en exigences [5]. Les consultations prénatales constituent l'un des 4 piliers de la maternité sans risque destinés à réduire la morbi-mortalité maternelle et périnatale [5-7].

Comme le suivi de grossesse recommandé diffère tant au niveau des termes et nombre de consultations qu'au niveau du type et de la fréquence des examens complémentaires de surveillance et de dépistage, il est difficile par conséquent de fournir une définition universelle d'un suivi de grossesse, qu'il soit adéquat ou insuffisant. L'Organisation Mondiale de la Santé (OMS) recommande au moins quatre visites prénatales [8]. Ce nombre diffère d'un pays à un autre selon les recommandations nationales de chaque pays. Il est de 11 aux Etats-Unis, de 10 en Allemagne et en Angleterre, de 9 en France et de 8 en Italie [9]. Le suivi prénatal est le plus souvent insuffisant en qualité et en nombre dans les pays en développement. Le taux de grossesses non ou mal suivies retrouvé est très variable, selon l'époque, le pays ou la région. Le taux de grossesses non suivies était de 38% en Centrafrique [10] et 32% au Kenya [11]. En RDC, les Enquêtes Démographiques et de Santé rapportaient que la proportion de femmes n'ayant pas reçu des soins prénatals était de 20% en 2007 et de 12% en 2014 [3]. Les grossesses non suivies se caractérisent par leur morbi-mortalité et maternelle et fœtale importante [12]. Une action préventive et des soins appropriés permettraient de réduire ces décès. Les consultations prénatales doivent permettre de mener la grossesse à bien pour la mère et son enfant surtout que la plupart des facteurs de risque liés à la grossesse peuvent être détectés au cours de celles-ci. Les programmes de la santé de la RDC prévoient des actions d'éducation pour la santé afin d'améliorer les connaissances et les attitudes des mères en matière de soins préventifs [3]. En d'autres termes, ces activités d'information et d'éducation portent sur l'intérêt et la nécessité des visites prénatales, de l'accouchement en milieu assisté, des visites post natales, de la planification familiale chez la mère et sur l'allaitement au sein, la nutrition, les soins corporels, la vaccination et la surveillance de la croissance chez l'enfant. Ces activités éducatives sont offertes aux femmes lors de tout contact avec les structures sanitaires publiques (centre de santé, maternité…) au cours des visites prénatales pendant leur grossesse, au moment de l'accouchement et au cours des visites post-natales.

L'une des cibles de l'objectif de développement durable 3 est de faire passer le taux mondial de mortalité maternelle au-dessous de 70 pour 100 000 naissances vivantes et celui de la mortalité néonatale à 12 pour 1 000 naissances vivantes au plus [2]. Et l'amélioration de santé de la mère et de l'enfant demeure encore un objectif prioritaire dans le monde, en particulier en RDC où des programmes de périnatalité ont été implantés dans le but de réduire la morbidité et la mortalité maternelle et infantile [13]. Etant donné que dans les milieux à ressources limitées comme le nôtre, où les décès maternels et périnatals restent parmi les plus élevés au monde, une prise en charge de qualité, en agissant en amont de l'accouchement et du post-partum pourrait contribuer à réduire le taux élevés de décès. C'est dans cet ordre d'idées que s'inscrit le présent travail qui vise à étudier les issues materno-fœtales des grossesses non suivies. L'intérêt accordé à ce sujet est motivé par la fréquence et la gravité des problèmes de santé dont souffrent la mère et son nouveau-né ainsi que les taux élevés de mortalité maternelle et néonatale enregistrés dans notre pays où l'accès aux soins obstétricaux et néonatals d'urgence demeure encore faible. En plus, les structures de soins, aussi bien de base que de référence, disposent de moyens d'intervention très limités. C'est ainsi que nous avons mené cette étude qui s'est fixé comme objectifs de déterminer la fréquence des grossesses non suivies à Lubumbashi, de décrire le profil sociodémographique des accouchées et d'évaluer la morbi-mortalité materno-fœtale lors de l'accouchement chez les femmes n'ayant pas suivi de consultations prénatales.

## Méthodes

**Type, cadre et période d'étude**: il s'agit d'une étude transversale analytique menée dans les maternités des 10 hôpitaux généraux de référence (HGR) de la ville de Lubumbashi en RDC (hôpital militaire de Ruashi, Cliniques Universitaires, hôpital Jason Sendwe, HGR Katuba, HGR Kenya, HGR Kamalondo, HGR Kisanga, HGR Kampemba, hôpital Gécamines-Sud et hôpital SNCC). Ces hôpitaux sont répartis dans les 7 communes que compte la ville de Lubumbashi. Elle était menée sur la période allant du 1^er^ décembre 2013 au 31 mai 2014.

**Figure 1:**
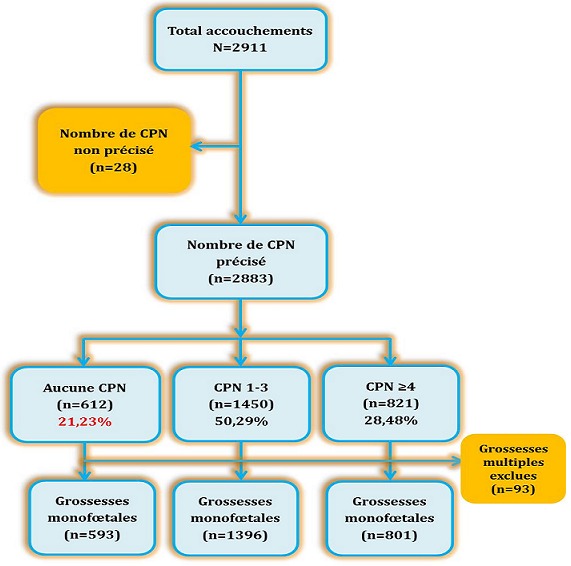
Distribution des accouchées enrôlées dans l´étude

**Population d'étude**: toutes les femmes qui se sont présentées dans ces formations sanitaires choisies pour un accouchement (grossesses ayant atteint au moins 22 semaines d'aménorrhée) ont été incluses de manière consécutive et exhaustive dans l'étude quel que soit le lieu de suivi de la grossesse. Le recrutement des sujets était exhaustif. Sur un total de 2911 accouchées enregistrées, le nombre de consultations prénatales avait été précisé chez 2883 d'entre elles. Après avoir exclu les grossesses multiples (n=93), la population étudiée (n=2790) a été regroupée selon le nombre de CPN en trois groupes: 1) **Groupe 1**: (consultations bien suivies) : si le nombre était supérieur ou égal à 4; 2) **Groupe 2**: (consultations prénatales non suivies) : lorsqu'aucun suivi de grossesse n'a été faite; 3) **Groupe 3**: (consultations mal suivies): si le nombre était compris entre 1 et 3. Dans les analyses de résultats, nous avons sélectionné comme groupe de référence, le groupe de femmes considérées comme bien suivies (CPN≥4) selon les recommandations de l'OMS qui fixent à 4 le nombre minimum de consultations prénatales qu'une femme doit bénéficier au cours d'une grossesse [8]. La figure 1 donne la distribution des accouchées enrôlées dans l'étude.

**Variables d'étude**: les caractéristiques sociodémographiques maternelles, les paramètres en rapport avec la morbi-mortalité maternelle et périnatale ont été recueillis par le personnel effectuant habituellement l'accouchement dans les sites d'enquête. Un entretien a permis de recueillir les caractéristiques sociodémographiques de la patiente ainsi que les antécédents obstétricaux. Une fiche d'enquête individuelle avait été élaborée à cet effet et la recherche de données complémentaires a été réalisée dans le dossier obstétrical pour la mère et néonatal pour le nouveau-né. Le nombre de consultations prénatales a été considéré comme variable dépendante. Les variables indépendantes étaient:

1) **caractéristiques sociodémographiques**: âge maternel, parité, niveau de scolarité, situation matrimoniale (femmes vivant seules des femmes vivant en union), profession (élève/étudiante, sans emploi et travailleuse ou ayant une activité rémunératrice).

2) **Paramètres en rapport avec la parturition, la morbidité et la mortalité maternelles**: mode d'admission (transférée et non transférée), état des membranes fœtales à l'admission (intactes et rompues), terme de la grossesse à la naissance (l'âge gestationnel est classé en <37,0 SA (prématurité), 37,0-42 SA (à terme) et >42 SA (post-maturité)), mode d'accouchement (césarienne ou voie basse), présentation fœtale (présentation céphalique de sommet et autres présentations ou mal présentations (front, siège, transverse, etc.)), utilisation de la solution ocytocique au cours du travail d'accouchement pour redynamiser celui-ci, épisiotomie, notion de transfusion sanguine, éclampsie, lésions de parties molles (réunissaient les déchirures cervicale, vaginale et périnéale), rétention placentaire, anémie (établie sur base des signes cliniques et/ou sur base d'un taux d'hémoglobine inférieur à 11 g/l quand cet examen était disponible), rupture utérine et issue maternelle (vivant ou décédé).

3) **Paramètres en rapport avec les nouveau-nés**: poids de naissance, dépression néonatale (appréciée par le score d'Apgar qui a été côté à la fin de la 5^ème^ minute après l'extraction du fœtus et est réparti en <7 (dépression néonatale) et en ≥7 (bon score)), transfert du nouveau-né dans le service de néonatologie, issue périnatale (nouveau-nés vivants et nouveau-nés décédés).

**Analyse des données**: le nombre de consultations prénatales est considéré ici comme variable dépendante et les paramètres en rapport avec la mère et le nouveau-né constituaient les variables indépendantes. Les caractéristiques sociodémographiques et la morbi-mortalité maternelle et périnatale des accouchées du groupe de CPN non suivies et celles du groupe de CPN mal suivies (1 à 3) ont été comparées à celles des accouchées du groupe de CPN bien suivies. Les fréquences sont présentées sous forme de pourcentages et les moyennes avec les écarts-types. Le test de X^2^ corrigé de Yates ou le test exact de Fisher ont été utilisés pour comparer les proportions. L'odds ratio (OR) a été calculé et présenté avec ses limites dans l'intervalle de confiance à 95% (IC à 95%) et le seuil de signification a été fixé à p<0,05. Les analyses ont été réalisées à l'aide du logiciel Epi Info 7.2.

**Considérations éthiques**: un consentement oral libre et éclairé de toutes les personnes impliquées dans cette étude a été obtenu verbalement. L'anonymat a été respecté. Les autorisations des Médecins Directeurs avaient été obtenues préalablement.

## Résultats

**Fréquence**: sur un total de 2883 accouchées consécutivement enregistrées au cours de la période d´étude et chez qui le nombre de CPN été précisé, 612 d'entre elles n'ont suivi aucune CPN, soit une fréquence de 21,23% (Figure 1). Le nombre moyen de CPN était 2,6 ± 1,9.

**Caractéristiques sociodémographiques des accouchées:** (Tableau 1) la proportion d'accouchées âgées de moins de 20 ans était de 5,37% dans le groupe 1 contre 11,97 et 7,59% respectivement dans les groupes 2 et 3. Quand on compare les proportions de moins de 20 ans entre les groupes 1 et 2, l'analyse statistique montre une différence significative signifiant que l'absence de suivi prénatal était 2,29 fois plus élevée chez les adolescentes que chez les femmes âgées de 20 à 34 ans (OR=2,29 [1,54-3,41]). Aucune différence significative n'a été retrouvée quant à la comparaison statistique entre les groupes 1 et 3. Les proportions de primipares et de grandes multipares étaient de 22,35% et 30,96% dans le groupe 1, de 19,39% et 34,23% dans le groupe 2 et de 19,56% et 35,60% dans le groupe 3. Quand on compare ces proportions de groupes 2 et 3 par rapport au groupe de référence, l'analyse statistique ne montre pas de différence significative. La proportion d'accouchées vivant seules était de 1,50% dans le groupe 1 contre 5,73 et 2,15% respectivement dans les groupes 2 et 3. Quand on compare les proportions de femmes vivant seules entre les groupes 1 et 2, l'analyse statistique montre une différence significative traduisant que l'absence de suivi prénatal était 4,0 fois plus élevée chez celles-ci que chez celles vivant en union (OR=4,00 [2,05-7,79]). Aucune différence significative n'a été retrouvée quant à la comparaison statistique entre les groupes 1 et 3. La proportion d'accouchées de bas niveau de scolarité (analphabète ou primaire) était de 10,86% dans le groupe 1 contre 33,22 et 14,83% respectivement dans les groupes 2 et 3. Quand on compare les proportions de femmes de bas niveau de scolarité entre les groupes 1 et 2, l'analyse statistique montre une différence significative traduisant que l'absence de suivi prénatal était 4,08 fois plus élevée chez celles-ci que chez celles ayant un niveau de scolarité élevé (secondaire/supérieur) (OR=4,08 [3,08-5,40]). De même, pour la comparaison entre les groupes 1 et 3 montre que la proportion de femmes de bas niveau de scolarité était significative élevée chez les femmes ayant suivi peu de CPN (OR=1,43 [1,09-1,86]). En répartissant les accouchées selon la profession, nous constations qu'il n'existe pas de différence statistiquement significative des différentes classes entre les groupes 1 et 2 (p>0,05). Concernant le groupe 3, la proportion de femmes sans profession étaient statiquement élevée comparée au groupe de référence signifiant que les femmes sans profession présentent 2,57 fois le risque de suivre peu de CPN en comparaison avec les femmes travailleuses (OR=2,57 [1,90-3,46]).

**Tableau 1:** Répartition des accouchées selon les caractéristiques socio-démographiques

Variable	Total (N=2790)	Groupe 1 (n=801)	Groupe 2 (n=593)		Groupe 3 (n=1396)	
	N	n (%)	n (%)	OR [IC95%]	n (%)	OR [IC95%]
**Age**						
<20 ans	220	43 (5,37)	71 (11,97)	2,29 [1,54-3,41]	106 (7,59)	1,43 [0,98-2,06]
20-34 ans	2081	603 (75,28)	435 (73,36)	1,00	1043 (74,71)	1,00
≥35 ans	489	155 (19,35)	87 (14,67)	0,78 [0,57-1,05]	247 (17,69)	0,92 [0,74-1,15]
**Parité**						
1	567	179 (22,35)	115 (19,39)	0,87 [0,66-1,16]	273 (19,56)	0,91 [0,73-1,14]
2-4	1275	374 (46,69)	275 (46,37)	1,00	626 (44,84)	1,00
≥5	948	248 (30,96)	203 (34,23)	1,11 [0,87-1,42]	497 (35,60)	1,20 [0,98-1,46]
**Statut matrimonial**						
Seule	76	12 (1,50)	34 (5,73)	4,00 [2,05-7,79]	30 (2,15)	1,44 [0,73-2,84]
En union	2714	789 (98,50)	559 (94,27)	1,00	1366 (97,85)	1,00
**Niveau de scolarité**						
Analphabète/ Primaire	491	87 (10,86)	197 (33,22)	4,08 [3,08-5,40]	207 (14,83)	1,43 [1,09-1,86]
Secondaire/ Supérieur	2299	714 (89,14)	396 (66,78)	1,00	1189 (85,17)	1,00
**Profession**	2790	801	593		1396	
Sans profession	2460	664 (82,90)	510 (86,00)	1,28 [0,92-1,77]	1286 (92,12)	2,57 [1,90-3,46]
Travailleuse	259	110 (13,73)	66 (11,13)	1,00	83 (5,95)	1,00
Etudiante	71	27 (3,37)	17 (2,87)	1,05 [0,53-2,07]	27 (1,93)	1,32 [0,72-2,42]

Comparaisons statistiques faites par rapport au groupe de référence (groupe 1)

**Environnement obstétrical:** (Tableau 2) la proportion de transférées était de 4,62% dans le groupe 1 contre 8,43 et 4,23% respectivement dans les groupes 2 et 3. Quand on compare les proportions d'évacuées obstétricales entre les groupes 1 et 2, l'analyse statistique montre une différence significative traduisant que l'évacuation obstétricale était 1,90 fois plus élevée chez celles n'ayant pas suivi de CPN (OR=1,90 [1,26-2,95]). Par contre, pour la comparaison entre les groupes 1 et 3 ne montre pas de différence significative. Du Tableau 3, nous constatons que les proportions de prématurité (18,98%) et de post-maturité (11,05%) étaient plus élevées dans le groupe 2 que dans le groupe 1 (11,26 et 8,57%) et le groupe 3 (14,35 et 9,20%). Quand nous comparons ces proportions entre les groupes 1 et 2, nous trouvons qu'il existe une association statistiquement significative entre la prématurité et l'absence de suivi prénatal et de même entre la post-maturité et l'absence de suivi prénatal; les risques pour les femmes n'ayant pas suivi de CPN de donner naissance à un prématuré ou un post-mature sont respectivement de 1,93 (OR=1,93 [1,33-2,80]) et 1,47 (OR=1,47 [1,00-2,30]). Quant à la comparaison entre les groupes 1 et 3, aucune différence statistique n'a été notée. La proportion de rupture des membranes fœtales à l'admission était de 20,85% dans le groupe 1 contre 25,63 et 21,92% respectivement dans les groupes 2 et 3. Quand on compare ces proportions entre les groupes 1 et 2, l'analyse statistique montre une différence significative traduisant que le manque de suivi prénatal exposait à 1,31 fois plus de risque d'être admise avec rupture des membranes (OR=1,31 [1,02-1,68]). Par contre, la comparaison entre les groupes 1 et 3 ne montre aucune différence statistique. La répartition des accouchées en fonction de la présentation fœtale montre que la mal présentation fœtale était notée dans 2,37%, 4,38% et 4,37% respectivement dans les groupes 1, 2 et 3. Quand on compare les proportions de malprésentation fœtale entre les groupes 1 et 2, l'analyse statistique montre une différence significative; les risques pour les femmes n'ayant pas suivi de CPN d'avoir un fœtus en malprésentation était de 1,89 (OR=1,89 [1,03-3,44]). De même, pour la comparaison entre les groupes 1 et 3 montre que la proportion de malprésentation fœtale était significative élevée chez les femmes ayant suivi peu de CPN (OR=1,88 [1,11-3,17]). Les proportions d'utilisation de solution ocytocique étaient de 20,10% dans le groupe 1, de 23,78% dans le groupe 2 et de 23,14% dans le groupe 3. Quand on compare ces proportions dans les groupes 2 et 3 par rapport celle du groupe de référence, l'analyse statistique ne montre pas de différence significative. La proportion de césarienne était de 6,24% dans le groupe 1 contre 10,62 et 7,88% respectivement dans les groupes 2 et 3. Quand on compare ces proportions entre les groupes 1 et 2, l'analyse statistique montre une différence significative traduisant que le manque de suivi prénatal exposait à 1,78 fois plus de risque d'accoucher par césarienne (OR=1,78 [1,21-2,63]). Par contre, la comparaison entre les groupes 1 et 3 ne montre aucune différence statistique. De ce tableau, nous constatons que la proportion d'épisiotomie était significative plus basse (7,59%) dans le groupe 2 que celle notée (12,23%) dans le groupe 1 traduisant que l'épisiotomie était moins pratiquée chez les femmes n'ayant pas suivi de CPN que chez celles qui les ont suivi (OR=0,59 [0,40-0,85]). Quant à la comparaison entre les groupes 1 et 3, aucune différence n'avait été trouvée.

**Tableau 2:** Répartition des accouchées selon l’environnement obstétrical

Variable	Total (N=2780)	Groupe 1 (n=801)	Groupe 2 (n=593)		Groupe 3 (n=1396)	
	N	n (%)	n (%)	OR [IC95%]	n (%)	OR [IC95%]
**Mode d’admission**						
Transférée	146	37 (4,62)	50 (8,43)	1,90 [1,26-2,95]	59 (4,23)	0,91 [0,59-1,39]
Non transférée	2644	764 (95,38)	543 (91,57)	1,00	1337 (95,77)	1,00
**Etat des membranes fœtales**						
Rompues	625	167 (20,85)	152 (25,63)	1,31 [1,02-1,68]	306 (21,92)	1,06 [0,86-1,32]
Intactes	2165	634 (79,15)	441 (74,37)	1,00	1090 (78,08)	1,00
**Présentation fœtale**						
Autre	106	19 (2,37)	26 (4,38)	1,89 [1,03-3,44]	61 (4,37)	1,88 [1,11-3,17]
Sommet	2684	782 (97,63)	567 (95,62)	1,00	1335 (95,63)	1,00
**Utilisation d’ocytocine**						
Oui	625	161 (20,10)	141 (23,78)	1,24 [0,96-1,60]	323 (23,14)	1,19 [0,97-1,48]
Non	2165	640 (79,90)	452 (76,22)	1,00	1073 (76,86)	1,00
**Mode d’accouchement**						
Césarienne	223	50 (6,24)	63 (10,62)	1,78 [1,21-2,63]	110 (7,88)	1,28 [0,90-1,82]
Voie basse	2567	751 (93,76)	530 (89,38)	1,00	1286 (92,12)	1,00
**Episiotomie**						
Oui	286	98 (12,23)	45 (7,59)	0,59 [0,40-0,85]	143 (10,24)	0,82 [0,62-1,07]
Non	2504	703 (87,77)	548 (92,41)	1,00	1253 (89,76)	1,00

Comparaisons statistiques faites par rapport au groupe de référence (groupe 1)

**Tableau 3:** Répartition des accouchées selon l’âge gestationnel

Age gestationnel	Total	Groupe 1	Groupe 2		Groupe 3	
	N	n (%)	n (%)	OR [IC95%]	n (%)	OR [IC95%]
Prématurité	265	67 (11,26)	67 (18,98)	1,93 [1,33-2,80]	131 (14,35)	1,34 [0,97-1,83]
A terme	1422	477 (80,17)	247 (69,97)	1,00	698 (76,45)	1,00
Post-maturité	174	51 (8,57)	39 (11,05)	1,47 [1,00-2,30]	84 (9,20)	1,12 [0,78-1,62]
Total	1861	595	353		913	

Comparaisons statistiques faites par rapport au groupe de référence (groupe 1)

**Morbidité et mortalité maternelles:** (Tableau 4) les proportions de lésions des parties molles étaient de 8,49% dans le groupe 1, de 7,60% dans le groupe 2 et de 6,16% dans le groupe 3. Quand on compare ces proportions dans les groupes 2 et 3 par rapport celle du groupe de référence, l'analyse statistique ne montre pas de différence significative. La proportion d'éclampsie était de 0,62% dans le groupe 1 contre 1,85 et 0,29% respectivement dans les groupes 2 et 3. Quand on compare ces proportions entre les groupes 1 et 2, l'analyse statistique montre une différence significative traduisant que le manque de suivi prénatal exposait à 3,0 fois plus de risque de présenter une éclampsie (OR=3,00 [1,09-8,70]). Par contre, la comparaison entre les groupes 1 et 3 ne montre aucune différence statistique. Les proportions de rétention placentaire étaient de 0,50% dans le groupe 1, de 0,67% dans le groupe 2 et de 0,43% dans le groupe 3. Quand on compare ces proportions dans les groupes 2 et 3 par rapport celle du groupe de référence, l'analyse statistique ne montre pas de différence significative. La proportion de rupture utérine était de 0,25% dans le groupe 1 contre 1,18 et 0,43% respectivement dans les groupes 2 et 3. Quand on compare ces proportions entre les groupes 1 et 2, l'analyse statistique montre une différence significative traduisant que le manque de suivi prénatal exposait à 4,76 fois plus de risque de faire une rupture utérine (OR=4,76 [1,00-47,19]). Par contre, la comparaison entre les groupes 1 et 3 ne montre aucune différence statistique. La proportion d'anémie était de 1,25% dans le groupe 1 contre 2,87 et 1,36% respectivement dans les groupes 2 et 3. Quand on compare ces proportions entre les groupes 1 et 2, l'analyse statistique montre une différence significative traduisant que le manque de suivi prénatal exposait à 2,33 fois plus de risque de faire une anémie (OR=2,33 [1,06-5,13]). Par contre, la comparaison entre les groupes 1 et 3 ne montre aucune différence statistique. Les taux de transfusion sanguine étaient de 0,87% dans le groupe 1, de 2,02% dans le groupe 2 et de 0,72% dans le groupe 3. Quand on compare ces proportions dans les groupes 2 et 3 par rapport celle du groupe de référence, l'analyse statistique ne montre pas de différence significative. Les proportions de décès maternel étaient de 0,12% dans le groupe 1, de 0,67% dans le groupe 2 et de 0,29% dans le groupe 3. Quand on compare ces proportions dans les groupes 2 et 3 par rapport celle du groupe de référence, l'analyse statistique ne montre pas de différence significative.

**Tableau 4:** Répartition des accouchées selon la morbidité et mortalité maternelles

Variable	Total (N=2790)	Groupe 1 (n=801)	Groupe 2 (n=593)		Groupe 3 (n=1396)	
	N		n (%)	OR [IC95%]	n (%)	OR [IC95%]
**Lésions des parties molles**						
Présentes	200	68 (8,49)	46 (7,60)	0,90 [0,61-1,34]	86 (6,16)	0,71 [0,51-1,08]
Absentes	2590	733 (91,51)	547 (92,40)	1,00	1310 (93,84)	1,00
**Eclampsie**						
Oui	20	5 (0,62)	11 (1,85)	3,00 [1,09-8,70]	4 (0,29)	0,46 [0,12-1,71]
Non	2770	796 (99,38)	582 (98,15)	1,00	1392 (99,71)	1,00
**Rétention placentaire**						
Oui	14	4 (0,50)	4 (0,67)	1,35 [0,25-7,29]	6 (0,43)	0,86 [0,24-3,06]
Non	2776	797 (99,50)	589 (99,33)	1,00	1390 (99,57)	1,00
**Rupture utérine**						
Oui	15	2 (0,25)	7 (1,18)	4,76 [1,00-47,19]	6 (0,43)	1,72 [0,30-17,50]
Non	2775	799 (99,75)	586 (98,82)	1,00	1390 (99,57)	1,00
**Anémie**						
Oui	46	10 (1,25)	17 (2,87)	2,33 [1,06-5,13]	19 (1,36)	1,09 [0,50-2,36]
Non	2744	791 (98,75)	576 (97,13)	1,00	1377 (98,64)	1,00
**Transfusion sanguine**						
Oui	29	7 (0,87)	12 (2,02)	2,34 [0,92-5,98]	10 (0,72)	0,82 [0,31-2,16]
Non	2761	794 (99,13)	581 (97,98)	1,00	1386 (99,28)	1,00
**Issue**						
Décès	9	1 (0,12)	4 (0,67)	5,43 [0,61-48,73]	4 (0,29)	2,29 [0,26-20,60]
Survie	2781	800 (99,88)	589 (99,33)	1,00	1392 (99,71)	1,00

Comparaisons statistiques faites par rapport au groupe de référence (groupe 1)

**Paramètres des nouveau-nés:** (Tableau 5) la répartition des nouveau-nés en fonction du poids de naissance montre que les proportions de faibles poids de naissance et de macrosomes étaient de 5,37 et 4,87% dans le groupe 1, de 11,64 et 4,72% dans le groupe 2 et de 6,45 et 6,88% dans le groupe 3. Quand nous comparons les proportions de faible poids de naissance entre les groupes 1 et 2, nous trouvons qu'il existe une association statistiquement significative entre le faible poids de naissance et l'absence de suivi prénatal; les risques pour les femmes n'ayant pas suivi de CPN de donner naissance à un faible poids de naissance est de 2,33 (OR=2,33 [1,56-3,46]). Quant à la comparaison entre les groupes 1 et 3, aucune différence statistique n'a été notée. La proportion de transfert en néonatologie était de 7,45% dans le groupe 1 contre 11,42 et 7,62% respectivement dans les groupes 2 et 3. Quand on compare ces proportions entre les groupes 1 et 2, l'analyse statistique montre une différence significative traduisant que le manque de suivi prénatal exposait à 1,60 fois plus de risque qu'un nouveau-né soit transféré en néonatologie (OR=1,60 [1,11-2,32]). Par contre, la comparaison entre les groupes 1 et 3 ne montre aucune différence statistique. La proportion de score d'Apgar déprimé (<7) était de 3,76% dans le groupe 1 contre 13,18 et 4,67% respectivement dans les groupes 2 et 3. Quand on compare ces proportions entre les groupes 1 et 2, l'analyse statistique montre une différence significative traduisant que le manque de suivi prénatal exposait à 3,89 fois plus de risque qu'un nouveau-né naisse déprimé (OR=3,89 [2,52-6,02]). Par contre, la comparaison entre les groupes 1 et 3 ne montre aucune différence statistique. La proportion de décès néonatal précoce était de 2,75% dans le groupe 1 contre 7,08 et 3,30% respectivement dans les groupes 2 et 3. Quand on compare ces proportions entre les groupes 1 et 2, l'analyse statistique montre une différence significative traduisant que le manque de suivi prénatal exposait à 2,7 fois plus de risque qu'un nouveau-né décède en période néonatale précoce (OR=2,70 [1,59-4,57]). Par contre, la comparaison entre les groupes 1 et 3 ne montre aucune différence statistique.

**Tableau 5:** Répartition des nouveau-nés selon les paramètres des nouveau-nés

Variable	Total	Groupe 1	Groupe 2		Groupe 3	
	N	n (%)	n (%)	OR [IC95%]	n (%)	OR [IC95%]
**Poids (grammes)**	(N=2790)	(n=801)	(n=593)		(n=1396)	
<2500	202	43 (5,37)	69 (11,64)	2,33 [1,56-3,46]	90 (6,45)	1,24 [0,85-1,81]
2500-3999	2425	719 (89,76)	496 (83,64)	1,00	1210 (86,68)	1,00
≥4000	163	39 (4,87)	28 (4,72)	1,04 [0,63-1,71]	96 (6,88)	1,46 [0,99-2,15]
**Transfert en néonatologie**	(N=2790)	(n=801)	(n=593)		(n=1396)	
Oui	229	59 (7,45)	65 (11,42)	1,60 [1,11-2,32]	105 (7,62)	1,02 [0,73-1,43]
Non	2510	733 (92,55)	504 (88,58)	1,00	1273 (92,38)	1,00
**Score d’Apgar**	(N=2739)	(n=792)	(n=569)		(n=1378)	
<7	173	30 (3,76)	78 (13,18)	3,89 [2,52-6,02]	65 (4,67)	1,26 [0,81-1,95]
≥7	2617	771 (96,24)	515 (86,82)	1,00	1331 (95,33)	1,00
**Issue**	(N=2790)	(n=801)	(n=593)		(n=1396)	
Décès	110	22 (2,75)	42 (7,08)	2,70 [1,59-4,57]	46 (3,30)	1,21 [0,72-2,02]
Survie	2680	779 (97,25)	551 (92,92)	1,00	1350 (96,70)	1,00
Total	2790	801	593		1396	

Comparaisons statistiques faites par rapport au groupe de référence (groupe 1)

## Discussion

Le taux de grossesses non suivies retrouvé est très variable, selon l'époque, le pays ou la région. Le taux de grossesses non suivies était de 21,23% dans notre étude, taux inférieur à ceux rapportés par Brown (au Kenya) [11], Fourn (au Bénin) [14] et Sepou (en Centrafrique) [10] qui avaient enregistré des fréquences respectives de 32, 33 et 38%. Dans une étude menée à Cotonou (Bénin), De Souza avait rapporté 50,7% de gestantes qui n'avaient pas fait de consultation prénatale [15]. Par contre, d'autres études rapportent des taux inférieurs au nôtre. Une étude menée au centre hospitalo-universitaire de Cocody (Côte d'Ivoire) avait enregistré 7,35% des gestantes qui n'avaient pas suivi de consultations [16]. Dans une étude faite à Bamako en 2007, Traoré rapporte un taux de 13,16% [12]. Apkadza, dans sa série sur l'accouchement sans surveillance médico-obstétricale rapporte 15,1% de femmes sans suivi prénatal [17]. A Marrakech (Maroc), El Hamdani avait trouvé que seulement 10% de femmes n'avaient pas suivi de consultations prénatales et avait souligné que la fréquence élevée d'utilisation des soins prénataux par les femmes enceintes de Marrakech s'expliquerait par plusieurs facteurs : le nombre important des centres de santé publique et bien répartis, la gratuité des services offerts dans ces centres, la présence de professionnel de santé uniquement féminin dans les services de soins maternels et l'impact positif des programmes de sensibilisation [18]. D'ailleurs, Prural avait noté que les femmes africaines utilisent largement les CPN lorsqu'elles sont accessibles [19]. Nous pensons que les variations des fréquences de l'absence de suivi prénatal sont fortement corrélées au niveau de développement des pays. Dans les pays en développement, les femmes ignorent souvent les avantages des soins prénatals pour la santé ou ne croient pas qu'ils sont importants pour elles [18]. Mais aussi, certains facteurs tels que les obstacles financiers et géographiques affectent l'utilisation par les femmes des services de soins de santé prénatals [20]. Le suivi des grossesses et leur déclaration ne sont pas toujours perçus comme indispensable pour les gestantes en raison de facteurs socioculturels intriqués et Ndiaye souligne l'intérêt du caractère multidisciplinaire des actions à mener pour y remédier [21].

Dans les pays développés, la grossesse non suivie existe et les taux d'absence de suivi prénatal varient de 1 à 3%. Elle n'a pas les mêmes problématiques que dans les pays en développement; l'accouchement ou le post-partum immédiat est pris en charge par des équipes qualifiées disponibles et les raisons de l'absence de suivi sont probablement différentes. Ces pays disposent de système de couverture maladie très opérationnel et d'un plateau technique ultraperformant qui facilitent un accès aux soins de santé et une prise en charge des gestantes [9]. L'analyse de la relation entre les consultations prénatales et les caractéristiques sociodémographiques des accouchées montre que l'absence de suivi était 2,29 fois plus élevée chez les adolescentes que chez les femmes adultes, 4 fois plus élevée chez les femmes vivant seules que chez celles vivant en union et 4,08 fois plus élevée chez les femmes de bas niveau de scolarité (analphabète ou primaire) que chez celles ayant un niveau de scolarité élevé (secondaire/supérieur). Ce constat est également retrouvé dans la littérature africaine [21-23]. Kakudji, dans une récente étude menée à Lubumbashi (RDC) trouvait une association très hautement significative entre l'adolescence et le manque de suivi prénatal [24]. Le profil sociodémographique des accouchées était très caractéristique de nos sociétés africaines. Outre leur jeune âge, elles sont célibataires, sans emploi et d'un bas niveau de scolarité avec une différence statistiquement significative. L'analphabétisme associé au refus du conjoint, à l'ignorance des risques et au caractère non désiré des grossesses étaient des facteurs supplémentaires décrits par Ndiaye [21]. Les femmes instruites sont plus aptes à assimiler des messages sur la santé maternelle et donc davantage susceptibles de consulter pour surveiller leur grossesse [25]. Fourn ajoutait que, dans certaines situations précises, les gestantes étaient retenues à domicile pour les activités ménagères [14]. Gandzien soulignait que la dépendance financière des gestantes vis-à-vis du conjoint ou de la famille était considérée comme un obstacle majeur au suivi des grossesses [23]. Enfin, d'autres aspects socioculturels retrouvés comme facteurs influençant le suivi prénatal sont les croyances selon lesquelles une grossesse connue tôt par l'entourage peut subir des mauvais sorts ou ne pas aboutir [10]. Les célibataires ont le plus souvent dissimulé leur grossesse à l'endroit de leurs parents, afin d'éviter les conflits familiaux [23]. Les raisons évoquées pour cette apathie vers les services de soins prénatals chez les jeunes femmes célibataires enceintes dans les pays en développement sont le manque de soutien familial ou social, les remarques désagréables des agents de santé et tentent de se soustraire du regard du public [26]. Une étude menée à Bulawayo avait montré que la crainte de dépistage du VIH constitue un facteur déterminant dans le manque de suivi de la grossesse chez les adolescentes [27]. Les jeunes femmes non mariées ont en effet un accès souvent difficile aux services de santé de la reproduction, ce qui explique leur faible utilisation de la contraception, notamment des méthodes modernes, et leur recours fréquent aux interruptions volontaires de grossesse. Comme ces dernières sont pratiquées le plus souvent dans des contextes non médicalisés, les risques de morbidité voire de mortalité sont élevés [26].

Notre étude n'a pas trouvé d'association significative entre la parité et l'absence de suivi prénatal. Par contre, Baumann [28] et Roth [29] avaient enregistré un nombre élevé de primipares. Au contraire, d'autres études ont observé que le taux de consultations prénatales diminue au fur et à mesure que la parité de la femme augmente et ce de manière significative [18, 30]. Ces dernières sont persuadées d'une bonne évolution de l'actuelle grossesse malgré l'absence de suivi prénatal, en raison du meilleur déroulement de la grossesse précédente malgré un suivi approximatif. Ces gestantes finissent par se convaincre de l'inutilité ou du peu d'intérêt des soins prénatals les encourageant à limiter la surveillance des grossesses ultérieures. Dans notre étude, les complications obstétricales et les issues de la grossesse pendant la période intra- et post-partales chez les femmes n'ayant pas suivi de consultations prénatales ont été comparées à celles les ayant suivis. Comparativement à ces dernières, nos résultats montrent que les femmes n'ayant pas suivi de CPN présentaient un risque élevé d'évacuation obstétricale, de rupture de membranes fœtales à l'admission, de malprésentation fœtale, d'accouchement par césarienne, d'éclampsie, d'anémie et de rupture utérine. Les taux de prématurité, de post-maturité, de dépression néonatale et de mortalité périnatale étaient significativement plus élevés chez les nouveau-nés issus de ces femmes. La rupture de membranes fœtales était fréquemment retrouvée chez les femmes sans suivi prénatal et ce de manière significative. Au cours des CPN, le dépistage et la prise en charge des infections cervico-vaginales sont faits. Chez les femmes ne bénéficiant pas de CPN, ces infections fragilisent les membranes fœtales qui par la suite vont se rompre et conduire à un accouchement prématuré. Rappelons que dans notre étude, nous avons noté significativement un taux élevé de prématurité chez celles-ci. La présente étude montre que le taux de césarienne était près de deux fois plus élevé chez les femmes non suivies comparativement aux femmes bien suivies, résultats similaires rapportés par plusieurs auteurs [10, 12] qui notaient que chez les femmes n'ayant bénéficié d'aucun suivi, la césarienne a été indiquée dans un contexte d'urgence, ce qui préjudiciable à la morbi-mortalité maternelle et fœtale. Le taux de césariennes élevé chez les femmes non suivies trouvé dans cette étude serait une conséquence de diverses complications de la grossesse et du travail (éclampsie, malprésentation fœtale, post-maturité) retrouvées plus fréquemment chez les non suivies que chez les bien suivies. De plus, les non suivies ont fréquemment été transférées d'une maternité à une autre.

D'où, beaucoup de gestantes en particulier les adolescentes non mariées n'ayant pas suivi de CPN et accouchent dans des structures de soins, parfois clandestines, peu équipées et ayant peu de compétences en soins obstétricaux et néonataux d'urgence. Les parturientes sont souvent alors référées dans les hôpitaux de niveau secondaire ou tertiaire en cas de complication ou lorsque le travail se prolonge, les membranes fœtales ayant préalablement été déjà rompues. En plus, les femmes non suivies voient le pronostic de leur accouchement se faire en salle de travail et parmi elles, celles qui avaient un bassin pathologique n'ont pas bénéficié de l'appréciation du bassin en fin de grossesse. L'appréciation du bassin aux dernières consultations prénatales permet d'évaluer le pronostic d'accouchement par voie basse et d'indiquer une césarienne élective beaucoup plus sécurisante. Ceci pourrait expliquer en partie le fait que nous ayons enregistré 4,76 fois de rupture utérine chez les femmes n'ayant bénéficié d'aucun suivi. Nos résultats concordent avec ceux de la littérature [10, 12]. Rasolonjatovo trouvait que 96,2% de femmes admises pour rupture utérine dans sa série n'avaient suivi aucune consultation prénatale [31]. La rupture utérine est une des complications la plus redoutable du travail et de l'accouchement. Elle engendre des conséquences graves tant maternelles que fœtales. Nous pensons que les ruptures utérines pourraient être conséquence d'une mauvaise gestion de la parturition dans les centres périphériques avec un plateau technique insuffisant et un personnel soignant non qualifié posant des actes inadéquats (usage abusif d'ocytociques, manœuvre de Chrsitellaire, non évaluation du risque en cas d'utérus cicatriciel etc.) dans le but lucratif. Toutes ces raisons contribuent à l'augmentation de taux de césariennes chez les femmes non suivies. Notre étude montre que les femmes sans suivi prénatal avaient un risque 2,33 fois plus de présenter une anémie en post-partum immédiat que celles qui étaient bien suivies. Ceci pourrait expliquer par plusieurs facteurs parmi lesquels nous pouvons citer le manque d'administration de la supplémentation martiale et du traitement préventif intermittent contre le paludisme pendant la grossesse mais aussi le taux élevé de complications intra-partales (rupture utérine, césarienne) chez ces femmes. Dans les régions endémiques pour le paludisme, l'anémie maternelle est une conséquence majeure du paludisme maternel [32]. Watson-Jones conclut que le paludisme et l'anémie maternels restent des causes importantes d'issues défavorables de la grossesse en Afrique subsaharienne [33]. L'OMS confirme l'effet bénéfique du traitement préventif intermittent à base de la sulfadoxine-pyriméthamine au cours des consultations prénatales et démontre que ce traitement réduit sensible le taux d'anémie maternelle chez les femmes qui en bénéficient [34]. Les issues néonatales pathologiques des grossesses constituaient encore une préoccupation, un véritable problème de santé publique dans tous les pays du monde [35]. En Afrique, près de 13 millions d'enfants meurent chaque année avant leur naissance ou tout juste après celle-ci [36]. Dans cette étude, le mauvais suivi des grossesses représentait un risque élevé d'accouchements prématurés comparé au groupe de femmes bien suivies. Le même constat avait fait déjà été fait dans la littérature [10, 23, 29] qui montrait que le manque de soins prénatals pouvait favoriser la poursuite de situations pathologiques car le risque de prématurité n'avait pu être recherché. Cette recherche des risques liés aux grossesses aurait permis de proposer des mesures préventives ou curatives [10].

Onze virgule soixante-quatre pourcent des nouveau-nés des femmes non suivies pesaient moins de 2500 grammes et le risque de naître avec un poids faible à la naissance était 2,33 fois plus élevé chez ces nouveau-nés que chez ceux issus des femmes bien suivies. Dans notre étude, les femmes n'ayant pas suivi les CPN donnaient plus significativement naissance aux FPN que celles qui en avaient suivi, ce qui est compatible avec les études menées à Lubumbashi et ailleurs [37-39]. L'étude de Fourn notait que trois quarts des enfants hypotrophiques étaient nés de mères qui n'avaient eu aucune visite prénatale et soulignait que cette dernière protègerait également contre l'hypotrophie [14]. Les soins prénatals appropriés sont importants dans la surveillance de la grossesse et la réduction des risques à la fois pour la mère et l'enfant pendant la grossesse et l'accouchement. Ils ont pour objectifs de prévenir, de dépister précocement et de prendre en charge les complications pouvant affecter la santé de la mère et de l´enfant à naître [19]. Les soins prénatals ont un effet protecteur direct sur le risque de survenue de FPN, comme démontré dans la présente étude et d'autres [40,41]. L'OMS recommande la mise en place de mesures de prévention parmi lequel un traitement préventif intermittent à base de la sulfadoxine-pyriméthamine au cours des consultations prénatales. L'importance de ce traitement a déjà été démontrée dans la réduction des taux de faible poids de naissance et de mortalité néonatale [34]. Le manque de suivi des grossesses a permis de laisser évoluer des situations pathologiques hors prise en charge médicale. Ainsi, le risque d'accouchements de faible poids de naissance ne pouvait être diminué chez ces femmes non suivies. La proportion de mauvais Apgar était statistiquement plus importante en cas d'absence de suivi prénatal dans notre étude. Ce constat était similaire à celui fait par d'autres auteurs [10, 12, 23]. L'absence de CPN présenterait alors des conséquences pour le nouveau-né avec un taux de prématurité élevé avec en corollaire le mauvais score d'Apgar. La mortalité néonatale précoce était près de trois fois plus élevée en cas de manque de suivi prénatal (7,08% vs 2,75% ; OR=2,70 [1,59-4,57]) dans notre étude. Plusieurs études antérieures retrouvaient des faits semblables [7, 10]. Ce taux de décès néonatals en cas de grossesses non suivies était proche à celui de Traoré qui avait observé 10,9% de décès néonataux [12]. Parmi les nouveau-nés issus de grossesses non suivies, 11,42% ont bénéficié d'un transfert en néonatologie. Le besoin de soins intensifs néonatals qui a été significativement enregistré chez les non suivies que chez les bien suivies serait secondairement dû au mauvais score Apgar. Mais aussi, ce mauvais pronostic périnatal qui a été considérablement noté dans le groupe de femmes non suivies serait attribuable aux taux élevés de complications anté- et intra-partales tels que l'évacuation obstétricale, l'éclampsie, l'anémie maternelle, la malprésentation fœtale, la rupture utérine et l'accouchement par césarienne, qui se sont produits fréquemment parmi les femmes qui n'ont bénéficié d'aucune CPN. Toutes ces complications ont une influence significative démontrée sur la morbidité et la mortalité périnatales.

**Limites de l'étude**: l'étude présente certaines limites. Les données recueillies concernant la fréquentation de CPN étaient pour une grande partie sur déclaration des accouchées bien que certaines ont présenté les carnets de CPN. L'évaluation de CPN s'est limitée au nombre de CPN sans entrer dans les différentes activités réalisées lors de ces CPN. Enfin, les accouchées ont été retenues indistinctement sans spécifier le caractère haut risque par rapport aux autres.

## Conclusion

La morbi-mortalité maternelle et périnatale constitue l'un des meilleurs indicateurs de l'état sanitaire d'une société et de son degré de développement. Cette étude montre que l'issue de grossesses non suivies constitue un problème majeur à Lubumbashi. Les déterminants de l'absence de suivi prénatal sont le fait qu'une femme soit jeune (<20 ans), qu'elle ne soit pas en union et qu'elle ait un bas niveau de scolarité (analphabète ou primaire). L'issue des grossesses est dominée par une forte morbidité maternelle et une morbi-mortalité périnatale élévée. C'est ainsi que nous recommandons aux autorités politico-sanitaires d'améliorer et de renforcer le système de santé en permettant l'accès aux soins de santé á moindre coût de la population et en particulier de la femme enceinte ainsi que d'évaluer les programmes déjà opérationnels en matière d'information et d'éducation portant sur l'intérêt et la nécessité des visites prénatales, de l'accouchement en milieu assisté. Une étude qualitative sous les consultations prénatales est à envisager car elle permettra d'assurer l'impact sur l'accouchement à Lubumbashi.

### État des connaissances actuelles sur le sujet

- L'absence de suivi prénatal constitue un problème majeur de santé publique en République Démocratique du Congo;
- Les grossesses non suivies se caractérisent par leur morbi-mortalité et maternelle et fœtale importante.

### Contribution de notre étude à la connaissance

- Aucune étude sur ce sujet n'a déjà été publiée antérieurement sur les facteurs de risque et le pronostic maternel et périnatal des grossesses non dans notre contexte, à Lubumbashi, République Démocratique du Congo;
- L'étude proposée est la première étude globale et multicentrique dans notre ville voire dans notre pays, intégrant une analyse factorielle permettant d'évaluer le pronostic maternel et périnatal dans notre contexte.

## Conflits d’intérêts

Les auteurs ne déclarent aucun conflit d'intérêt.
